# Micronutrient status, food security, anaemia, *Plasmodium* infection, and physical activity as predictors of primary schoolchildren's body composition in Côte d'Ivoire

**DOI:** 10.3389/fnut.2024.1524810

**Published:** 2025-01-29

**Authors:** Kurt Z. Long, Sylvain G. Traoré, Kouadio B. Kouassi, Jean T. Coulibaly, Bomey C. Gba, Daouda Dao, Johanna Beckmann, Christin Lang, Harald Seelig, Nicole Probst-Hensch, Uwe Pühse, Markus Gerber, Jürg Utzinger, Bassirou Bonfoh

**Affiliations:** ^1^Swiss Tropical and Public Health Institute, Allschwil, Switzerland; ^2^University of Basel, Basel, Switzerland; ^3^Université Peleforo Gon Coulibaly, Korhogo, Côte d'Ivoire; ^4^Centre Suisse de Recherches Scientifiques en Côte d'Ivoire, Abidjan, Côte d'Ivoire; ^5^Université Nangui Abrogoua, Abidjan, Côte d'Ivoire; ^6^Université Félix Houphouët-Boigny, Abidjan, Côte d'Ivoire; ^7^Department of Sport, Exercise and Health, University of Basel, Basel, Switzerland

**Keywords:** body composition, micronutrients, anaemia, food insecurity, *Plasmodium falciparum*

## Abstract

**Background:**

Stunting and overt malnutrition remain prevalent among school age children in rural areas of Côte d'Ivoire while obesity is increasing in urban areas. Associations of children's nutritional status, *Plasmodium* infection, physical activity and household characteristics with body composition were analyzed to identify what factors might be contributing to this dual burden of disease.

**Methods:**

Longitudinal growth curve models (LGCM) evaluated associations of micronutrient status, household food security, *Plasmodium falciparum* prevalence and physical activity assessed at three time points with fat free mass and fat mass.

**Results:**

More severe anaemia was inversely associated with FFM and TrFFM trajectories overall and among girls. *P. falciparum* infection had an indirect inverse association with FFM trajectories through anaemia among girls and through reductions of vitamin A directly associated with FFM. Changes in zinc concentrations were positively associated with FM trajectories overall and among boys. Food insecurity was inversely associated with FFM among boys from lower socio-economic status (SES) households while increased MVPA was associated with reduced fat mass among girls.

**Conclusions:**

The integration of Malaria control programs with efforts to improve household healthy diet and promote physical activity can lead to improvements in body composition and overall child health and wellbeing.

## Graphical Abstract

Micronutrient status, dietary diversity, food security, infections with *Plasmodium falciparum*, anaemia and physical activity as predictors of primary schoolchildren's body composition in Côte d'Ivoire. 
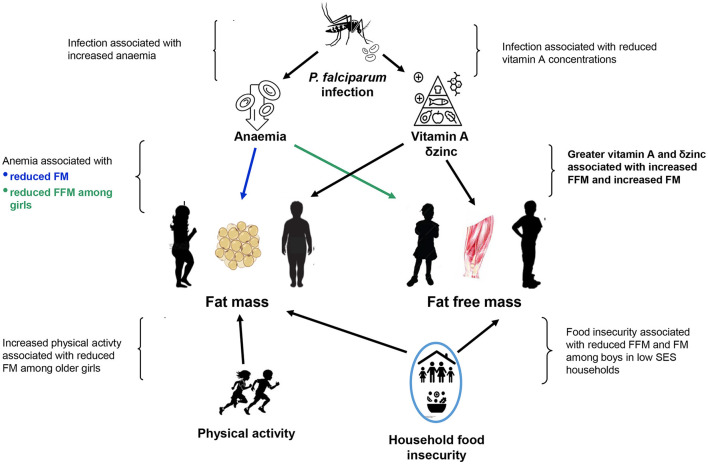

## 1 Introduction

Patterns of childhood disease burden are increasingly divergent across different regions of Sub-Saharan Africa (SSA). Childhood stunting and overt malnutrition are prevalent in more rural areas while childhood overweight and obesity is now increasing in urban settings (1). This dual burden of disease is compounded by the continued high prevalence of infectious diseases such as malaria. These trends have led to contrasting underlying patterns of fat mass and muscle mass among school-age children, which can determine long-term risk of chronic metabolic diseases and comorbidities.

Such patterns and trends are present among school-aged children in West Africa. A study carried out in Dakar, Senegal reported an overall prevalence of obesity of 9.34%, with 2.88% of boys and 6.46% of girls being classified as obese (2). Seven percent of schoolchildren from urban areas of Lomé, Togo were found to be overweight/obese while 18% were underweight (3). In Côte d'Ivoire, 9% of schoolchildren living in Abidjan were overweight or obese, whereas 65% were thin or extremely thin (4). A recent literature review of studies conducted in Côte d'Ivoire also found a high prevalence of under nutrition, along with emerging overweight and obesity (5). Nutrition health policies in Côte d'Ivoire concerned with these issues are principally focused on U5 stunting and wasting, low birth weight and, to a lesser extent U5 overweight (6).

Differences in household socio-economic status (SES) and food security maybe contributing to these disparate nutritional patterns among children. Rural households in Côte d'Ivoire may not have the economic means to provide a diverse, healthy diet for their children leading to poor growth and development (7). Low SES household may also contribute to the low prevalence of overweight and obesity. This could have delayed the onset of the nutrition transition seen in many low- and middle-income countries (LMICs) where traditional diets high in cereal and fiber have been replaced by more Western pattern diets rich in sugars, fat, and animal-source food (8).

Underlying changes in micronutrient status, physical activity (PA) and infectious diseases may contribute to these changing patterns on body composition. Vitamin and mineral deficiencies are associated with obesity and greater fat mass in adults and children (9, 10), Supplementation trials have reported that carotenoids and derivatives as well as vitamin D can reduce body mass index (BMI) z-scores and fat mass accrual among overweight and obese children and adults (11, 12). Micronutrient status also plays a role in muscle mass development with inverse relationships found between serum iron and vitamin D status and muscle mass and regeneration (13, 14).

PA is associated with reduced overweight, obesity, waist circumference (15) and higher muscle mass (16). Among children in sub-Saharan Africa, lower PA is associated with higher overweight and obesity, higher relative body fat, and lower relative fat-free mass (17). Intervention trials carried out in disadvantaged primary schools in South Africa reported that increased PA levels are associated with decreases in children's body mass index and lower risks of obesity (18).

The prevalence of malaria continues to be high in West Africa and Côte d'Ivoire with *P. falciparum* the predominant species accounting for 95% of all cases (19). Malaria and asymptomatic infections can result in a greater prevalence of anaemia and iron (Fe) deficiency especially among infant and young children infants (20). Treatment of malaria among school-aged children has also been found to decreases anaemia across transmission settings (21). Malaria and *P. falciparum* infections may have indirect effects on body composition through their effects on anaemia.

Few studies have simultaneously addressed the relative roles of micronutrient status, PA, infectious diseases and household factors in determining body composition outcomes (22). As a result, there is still an incomplete understanding of how these biological and household factors interact to determine outcomes. As such, it is not clear if different factors have primarily direct effects on body composition or indirect effects through single or multiple pathways, which has important implications for the efficacy of public health interventions. The clarification of these relationships can contribute to the development of more comprehensive preventive strategies that improve school-age children's body composition, health and wellbeing.

The continued prevalence of childhood malnutrition in Côte d'Ivoire and the emergence of overweight and obesity present a challenge in efforts to improve school-age children's nutrition and health. The current study carried out a longitudinal analysis of children's body composition in Côte d'Ivoire in an effort to clarify the relative roles and interactions of micronutrient status, PA, infectious diseases and household factors in determining these outcomes (23). The overall objective is to evaluate associations of estimated changes in fat free mass (FFM) and fat mass (FM) with the three groups of variables hypothesized to impact body composition: (1) serum micronutrient concentrations, anaemia, dietary diversity and household food insecurity, (2) *P. falciparum* and STH infections, and (3) accelerometer-measured PA. It will also test how *P. falciparum* infections are indirectly associated with body composition through their effects on children's micronutrient status and anaemia. Understanding how the interactions of these different factors determine body composition patterns among pre-adolescent children can aid in the development of more effective community-specific interventions that reduce childhood obesity and long-term metabolic disease in rapidly urbanizing Sub-Saharan countries.

## 2 Materials and methods

### 2.1 Study population

Children included in this analysis were part of a larger cluster-randomized, double-blind, placebo-controlled trial assessing effects of PA and/or multi-micronutrient supplementation (MMNS) on PA/fitness, micronutrient status, body composition/obesity, infectious disease, and cognitive function in public primary schools located in Gqeberha (South Africa), Ifakara (Tanzania), and Taabo (Côte d'Ivoire). In the Taabo component of the study, children were recruited from eight public primary schools randomly selected in Taabo Health and Demographic Surveillance System (HDSS) in South-central Côte d'Ivoire (24). Schools were eligible if they were not involved in any other research projects of this nature, had facilities to implement physical education lessons, and not participating in government nutrition programs.

### 2.2 Participants, procedure and change in study design due to a national teacher strike and the COVID pandemic

Children were enrolled if they were 6–12 years old at baseline, attended grades 1–4, did not have clinical conditions preventing participation in physical education lessons, were not participating in other research projects or clinical trials and not participating in any food/nutritional program. Children were excluded from data analyses (but not from the intervention) if they had gastro-intestinal tract alterations impairing MMNS absorption or had received food/micronutrient supplements in the past 6 months. Power calculations for the overall study indicated that a total sample of 1,096 children was needed per study site (G^*^power 3.1: *f* = 0.10, alpha error probability = 0.05, power = 0.80, number of groups = 12, number of measurements = 3). Assuming an overall dropout-rate of 10%, the targeted sample size was determined to be 1,320 children per country with 330 children assigned to one of the four intervention arms. Enrolled children were assigned an identification number after obtaining parent/guardian granted written informed consent and children's oral consent.

Children were recruited class-wise from public schools. Classes were initially randomly assigned to one of four groups: (a) a physical activity + placebo group (PA); (b) a multi-micronutrient supplementation group (MMNS); (c) a physical activity + multi-micronutrient supplementation group (PA + MMNS); and (d) a placebo control group. Randomization ensured that all four-intervention arms were present in each school across the four grade levels with balanced age groups. Project coordinators generated the random allocation sequence while local project coordinators enrolled participants by classes to interventions.

For the MMNS arm, it was anticipated that children would receive a daily tablet based on the MixMe^TM^ powder sprinkle (DSM, Basel, Switzerland) found to reduce micronutrient deficiencies and improve weight-for-age z-scores among South African children. Vitamin A was replaced with 4,500 mg of β-carotene in these tablets based on findings that carotenoids reduce BMI z-scores and abdominal adiposity accrual among obese children (11). Children in the PA and non-intervention arms were expected to receive a placebo product similar in packaging and taste to ensure children, teachers, and study personnel were blinded. Teachers administered the MMNS and placebo tablets 5 days per week to the schoolchildren.

Data assessments were carried out at children's schools at baseline (T1, October to December 2018), at T2 (January to March 2020) and at T3 (January to March 2021). A national teacher strike began after the baseline data assessment and ended before the T2 assessment. The COVID19 pandemic broke out shortly after the start of the interventions resulting in temporary school closures, suspension of physical education lessons, and loss of children to follow-up, and so affected the implementation of the T3 assessment. These interruptions also led to intermittent implementation of the MMNS intervention and the non-implementation of the PA intervention. Consequently, the present study is treated as longitudinal analysis with no comparisons made between students assigned to the different intervention arms.

### 2.3 Body composition

The primary study outcomes were FFM, FM, truncal fat free mass (TrFFM) truncal fat mass (TrFM) assessed via bioelectrical impedance analysis (BIA) using a wireless body composition monitor (Tanita MC-580; Tanita Corp., Tokyo, Japan). The MC-580 was also used to assess bodyweight, to the nearest 0.1 kg. Height was measured to the nearest 0.1 cm with each child standing with his/her back erect and shoulders against a stadiometer. Sex-specific height, and weight-for-age and BMI z-scores were computed from the CDC/WHO growth reference data (25). Children with height z-scores < −2 SD were classified as stunted. Children were also classified as underweight (< 5th percentile), normal weight (5th−84th percentile), overweight (85th−94th percentile), and obese (>95th percentile).

### 2.4 Moderate-to-vigorous physical activity

Children's PA was assessed objectively using an accelerometer. The accelerometry device (Actigraph wGT3x-BT, Shalimar, FL, USA), previously validated with children (26, 27), was worn around the hip for 7 consecutive days to assess a full weekly period, with a sampling epoch of 15 s (28). Time per day spent in moderate-intensity PA (MPA) (>3 MET) [metabolic equivalents of task] and vigorous-intensity PA (VPA) (>6 MET) was determined based on the raw accelerometry counts and the ActiLife? computer software, with cut-off values derived from Freedson et al. (29). Children were included if they had a minimum of five valid days (4 weekdays, 1 weekend day) with at least 8 h of wear-time per day (30).

### 2.5 Micronutrients and anaemia evaluation

Micronutrient concentrations were determined from five 50 μL volumes of blood collected from children at the T1, T2 and T3 assessment points and spotted onto dried blood spots (DBS) cards. Eluted blood spots were screened for concentrations of retinol binding protein (RBP), a marker of vitamin A, vitamin D, zinc, and serum transferrin receptor (sTfR), a marker of iron deficiency (31), by the Neuberg Global Laboratories (Durban, South Africa). Briefly, commercial sandwich-ELISA kits for sTfR (Elabscience, USA), vitamin D (Euroimun, Germany) and RBP (Arbor Assays, USA) were used where samples were added to micro plate wells pre-coated with an antibody for the specific marker followed by addition of biotinylated detection antibody for the marker and Avidin-Horseradish Peroxidase (HRP) conjugate and reactions read according to kit instructions. For the zinc assay zinc in the sample was chelated with 5-Bromo-2-pyridylazo (5-BR-PAPS) and then read (Biorex diagnostics, UK). Hemoglobin (Hb) concentration were measured with a HemoCue^®^ Hb 301 system according to the manufacturer's instructions. Children were classified as non-anaemic, mildly anaemic, moderately anaemic and severely anaemic using WHO hemoglobin cut-off points (32).

### 2.6 Dietary diversity and food security

A culturally sensitive food frequency questionnaire (FFQ) administered to the child's carer at baseline, identified 149 food items consumed by the child's within the past 24 h using the Food and Agriculture Organization questionnaire (33). An aggregated Dietary Diversity Score (DDS) for each child was created by categorizing the reported food items into 12 food groups, transformed into dummy variables (1 = yes, 0 = no) and summing the number of these reported food groups. Food insecurity was measured using a questionnaire based on the Household Food Insecurity Access Scale (34). Caregivers were asked if: In the past 30 days, (i) was there no food to eat of any kind in your house due to lack of resources, (ii) did any household member go to sleep at night hungry because there was not enough food, and (iii) did any household member go a whole day and night without eating? Summed answers ranged from a score of 0 indicating good access to food (household food security) and 6 indicating prevalence of hunger (household food insecurity). Children's caregivers were also interviewed at baseline to determine children's personal characteristics and family SES.

### 2.7 *P. falciparum* and STH prevalence

A stool sample (at least 15 g) was collected from children 24 h following data collection in a pre-labeled (unique ID and name) stool container and transported to a laboratory for diagnostic work-up within the same day. The Kato-Katz technique was used for the detection of STH infection (*A. lumbricoides, T. Trichiura* and hookworms) (35). For each sample, a duplicate 41.7 mg thick smear was prepared and read under a microscope by laboratory technicians from Center Suisse de Recherches Scientifiques en Côte d'Ivoire (CSRS). STH infection intensity (light, moderate, heavy) for each species was calculated based on the numbers of helminth eggs per gram (EPG) (36). Children were tested for *P. falciparum* infections using a Rapid Malaria Test.

### 2.8 Statistical methods

Means of the study endpoints of FFM, TrFFM, FM and trFM and the percentage of children who were stunted, wasted and overweight at baseline were first calculated and then compared between sexes using chi-square tests for categorical variables and *t*-tests for continuous variables.

Associations of body composition trajectories with the independent variables were evaluated using longitudinal growth curve models (LGCM). LGCM is a form of longitudinal analysis that estimates growth trajectories within the framework of structural equation models (SEM). It can model a unique trajectory for each subject along with a mean trajectory for all individuals and can also control for measurement error with multiple indicators of latent variables. For this analysis the separate body composition outcomes were first modeled as a latent variable of change defined from the T1, T2 and T3 measures with random intercept representing initial status and slope(s) of the growth rate trajectory over time. The growth trajectories were then regressed on micronutrient concentrations at single time points, changes in concentrations (defined as T3 concentrations minus T1 concentrations) and anaemia to determine their role in regulating fat and muscle mass development. Baseline DDS and household food insecurity scores and their interactions with household SES were included in models to ascertain if disruption of household food intake and eating patterns due to lack of resources is associated with body composition trajectories. Associations of *P. falciparum* and STH infection intensity with body composition were then tested to determine the effects of infections on body composition changes. Finally, MVPA estimates and a MVPA-age interaction term were included to determine the effect of energy expenditure on each body composition trajectory by age.

The indirect effects *P. falciparum* and STH infections on body composition changes when mediated by children's micronutrient status was assessed by regressing the disease variables on micronutrient concentrations and anaemia that had been directly regressed on the body composition trajectories (paths α and β in Figure 2, respectively). The product of the two path coefficients was used to calculate indirect mediated effects when the two paths were statistically significant (α^*^β). Models were run for all children combined and stratified by sex.

The intercept and body composition trajectories were regressed on children's baseline height-for-age z-scores to control for initial stature differences. A school class random intercept was included in the LGCM to account for the nested (clustered) nature of the data. Model estimation was based on a robust maximum likelihood estimator (MLR) with missing data handled via full information maximum likelihood (FIML). Model chi-square statistics, Comparative Fit Index (CFI), Tucker-Lewis Index (TLI) and the Root Mean Square Error Approximation (RMSEA) were used to assess model fit (4). Indirect, direct, and total effects of antecedents upon body composition were calculated using the MODEL CONSTRAINT: subcommand in Mplus.

## 3 Results

### 3.1 General characteristics

Overall, 1,358 children from grades 1–4 (aged 5–12 years) were enrolled from eight public primary schools in Taabo-Cité and Taabo-Village. Estimates of FM, FFM, TrFM and TrFFM were collected from 958 children at baseline (T1, October to December 2018) due to illness, absence from school or refusal to participate (Supplementary Figure 1). Subsequently, 885 children were assessed at T2 the second measurement (January to March 2020) and 777 children at T3 (January to March 2021). Serum concentrations of RBP, zinc, vitamin D and sTfR were derived from 998 DBS collected from children at T1, 790 DBS from children at T2 and 580 DBS samples from children at T3. Overall, the average age of children was 8.35 with no differences between the sexes (Supplementary Table 1). Girls had higher FM and TrFM compared to boys while boys had higher TrFFM compared to girls at baseline.

### 3.2 Fat free mass

**Figure 1:**
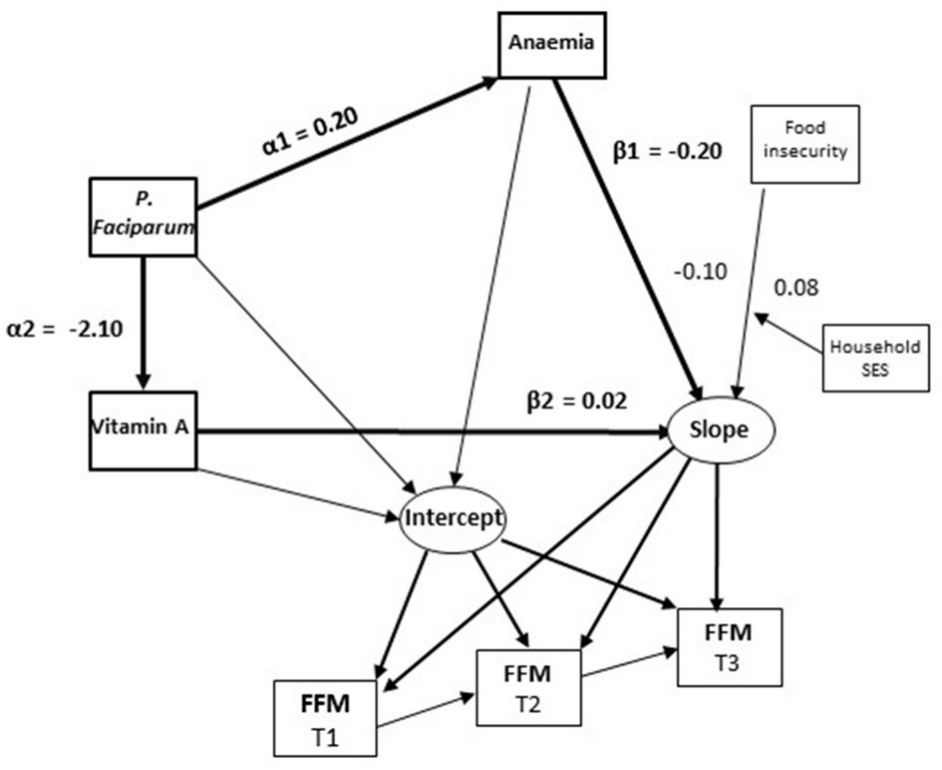
Associations of micronutrient status, *P. falciparum* prevalence and child characteristics with changes in fat-free mass (FFM) in Côte d‘Ivoire. Paths in bold (α1*β1 and a2*b2) represent statistically significant indirect effects of *P. falciparum* on FFM changes mediated by anaemia and vitamin A.

More severe anaemia was inversely associated with the trajectory of FFM while vitamin A concentrations were positively associated with this trajectory (Table 1; Figure 1). *P. falciparum* infection at T2 was positively associated with anaemia and so had an indirect inverse association with FFM trajectory (Table 1; α1^*^β1 in Figure 1). *P. falciparum* infection also had an indirect inverse association with the FFM trajectory resulting from its inverse association with vitamin A concentrations (α2^*^β2 in Figure 1). No direct associations were found between FFM trajectories and measures of dietary diversity and food insecurity.

**Table 1:** Associations of micronutrient status, household characteristics and *P. falciparum* prevalence with changes in fat free mass (FFM) among 958 children in Côte d‘Ivoire overall and by sex.

	**Overall** ^**a**^		**Boys**		**Girls**	
	**FFM**	**95% CI**	**FFM**	**95% CI**	**FFM**	**95% CI**
**Changes in FFM (T1-T3)** ^b^						
Anaemia T3	**−0.20**	**(−0.31**, **−0.06)**	−0.16	(−0.32, 0.02)	**−0.26**	**(−0.43**, **−0.10)**
Vitamin A concentrations T2	**0.02**	**(0.01, 0.03)**	−0.02	(0.01, 0.03)	0.01	(−0.14, 0.30)
Household food insecurity	−0.10	(−0.95, 0.75)	**−2.45**	**(−4.03**, **−0.88)**	0.14	(−0.10, 1.21)
Household SES	−0.17	(−1.02, 0.66)	**−3.56**	**(−5.40**, **−1.74)**	0.35	(−0.60, 1.20)
SES^*^food insecurity	0.08	(−0.20, 0.36)	**1.20**	**(0.60, 1.81)**	−0.10	(−0.42, 0.23)
***P. falciparum*** **effect on micronutrient mediators**						
**Anaemia T3**						
*P. falciparum* T2	**0.20**	**(0.08, 0.32)**	0.01	(−0.10, 0.03)	**0.27**	**(0.11, 0.42)**
**Vitamin A concentrations T2**						
*P. falciparum* T2	**−2.10**	**(−2.90**, **−1.26)**	**−2.15**	**(−3.60**, **−0.81)**	**−1.91**	**(−3.00**, **−1.02)**
**Indirect effects of** ***P. falciparum*** **on FFM**^c^						
*P. falciparum* mediated by anaemia	**−0.04**	**(−0.32**, **−0.01)**	−0.06	(−0.02, 0.08)	**−0.08**	**(−0.13**, **−0.01)**
*P. falciparum* mediated by vitamin A	**−0.04**	**(−0.05**, **−0.01)**	−0.05	(−0.10, 0.01)	0.01	(−0.05, 0.03)

**Figure 2:**
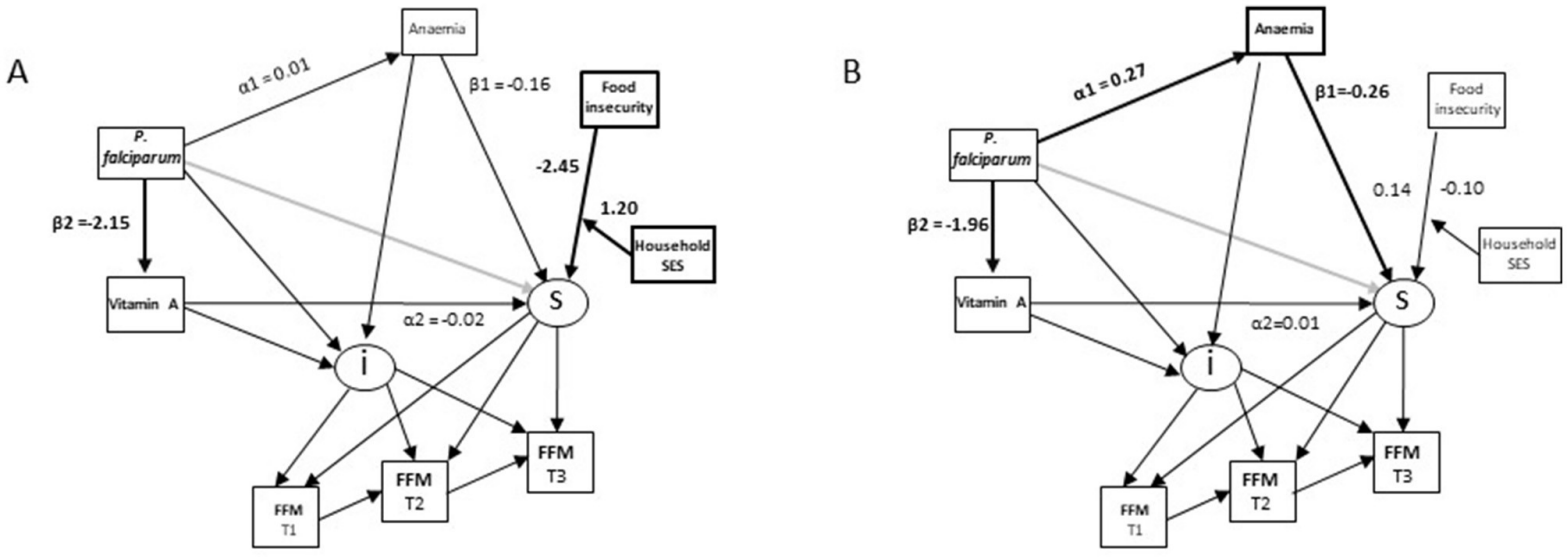
Differences in associations of micronutrient status, *P. falciparum* prevalence and child characteristics with changes in fat-free mass (FFM) in Côte d‘Ivoire by sex. **(A)** boys. **(B)** girls.

Boldface indicates statistical significance (*p* < 0.05). ^a^Model includes gender and baseline age. ^b^Data are presented as slope values for FFM (95% CI) estimated across three data collection rounds growth curve models. ^c^Estimated indirect effects of *P. falciparum* on FFM trajectory mediated by anaemia (α1^*^β1 in Figure 2) and vitamin A (a2^*^b2).

An inverse association of anaemia with FFM was again found among girls (Table 1; Figure 2). *P. falciparum* infection again had an indirect inverse association with FFM trajectory through anaemia (α1^*^β1 in Figure 2). These associations were not found among boys. An interaction term between household food insecurity and household SES was positively associated with FFM in the model for boys suggesting that food insecurity was associated with greater reductions in FFM among boys from low SES households. This term was not significant in the model for girls (Table 1).

### 3.3 Fat mass

**Figure 3:**
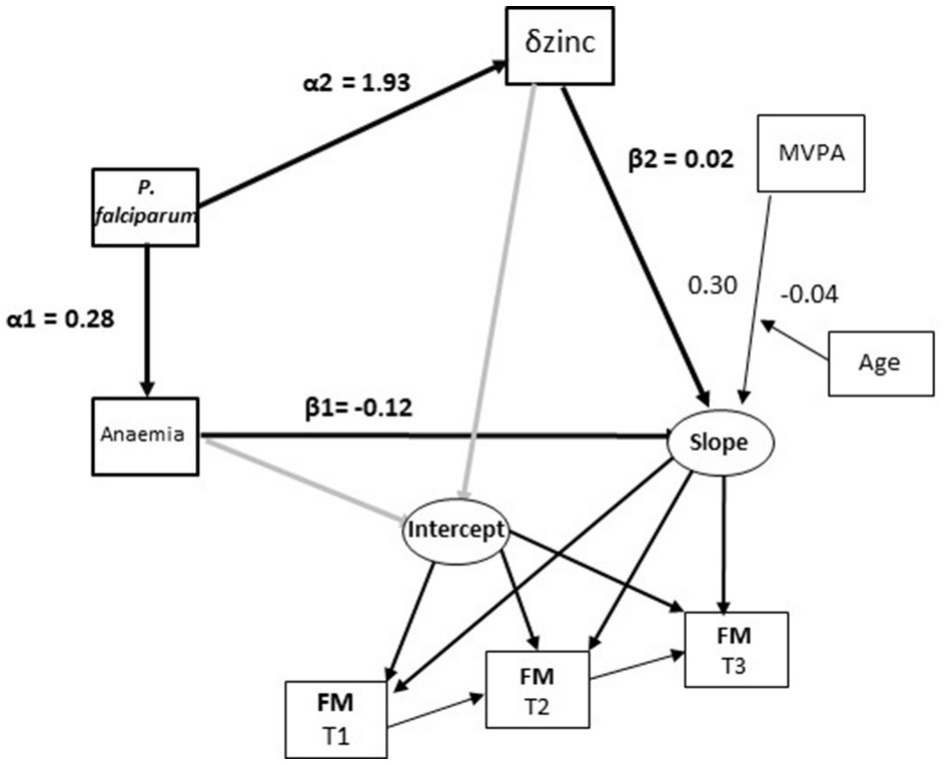
Associations of micronutrient status, *P. falciparum* prevalence and, child characteristics with changes in fat mass in Côte d‘Ivoire. Paths in bold (α1*β1 and a2*b2) represent statistically significant indirect effects of *P. falciparum* on FM changes mediated by anaemia and change in zinc status.

Anaemia was also inversely associated with the FM trajectory while vitamin A and increases in zinc concentrations from T1 to T2 were positively associated with this trajectory (Table 2). *P. falciparum* infections again had an indirect inverse association with FM when mediated by anaemia (Table 2; α1^*^β1 in Figure 3). *P. falciparum* infection was also positively associated with zinc concentrations changes and so now had an indirect positive association with the FM trajectory (Table 2; α2^*^β2 in Figure 3).

**Table 2:** Associations of micronutrient status, household characteristics and *P. falciparum* prevalence with changes in fat mass (FM) among 958 children in Côte d‘Ivoire^a^ overall and by sex.

	**Overall** ^**a**^		**Boys**		**Girls**	
	**FM** ^b^	**95% CI**	**FM**	**95% CI**	**FM**	**95% CI**
**Change in FM (T1– T3)** ^b^						
Anaemia T3	**−0.12**	**(−0.20**, **−0.04)**	−0.10	(−0.21, 0.16)	−0.09	(−0.24, 0.01)
Change in zinc concentrations T1-T3	**0.02**	**(0.01, 0.03)**	**0.02**	**(0.01, 0.03)**	0.01	(−0.01, 0.02)
Vitamin A T1	**0.02**	**(0.01, 0.03)**	0.02	(−0.01, 0.04)	0.01	(−0.01, 0.03)
Household food insecurity	0.32	(−0.60, 0.64)	−0.93	(−1.96, 0.93)	0.33	(−0.43, 1.10)
Household SES	0.06	(−0.55, 0.66)	**−1.20**	**(−2.36**, **−0.01)**	0.40	(−0.27, −1.11)
SES^*^food insecurity	−0.01	(−0.20, 0.20)	**0.41**	**(0.12, 0.80)**	−0.11	(−0.34, 0.12)
Age	**0.18**	**(0.12, 0.24)**	0.04	(−0.05, 0.13)	**0.27**	**(0.21, 0.34)**
MVPA	0.32	(−0.12, 0.72)	−0.13	(−0.74, 0.40)	**0.70**	**(0.11, 1.30)**
Age^*^MVPA	−0.05	(−0.10, 0.01)	0.02	(−0.04, 0.10)	**−0.08**	**(−0.15**, **−0.02)**
***P. falciparum*** **effect on micronutrient mediators**						
**Anaemia T3**						
*P. falciparum* T2	**0.28**	**(0.13, 0.43)**	**0.36**	**(0.13, 0.60)**	**0.20**	**(0.03, 0.34)**
**Change in zinc concentrations**						
*P. falciparum* T2	**1.93**	**(0.71, 3.16)**	**3.00**	**(0.82, 4.40)**	1.55	(−0.16, 3.22)
**Indirect effects of** ***P. falciparum*** **on FM**^c^						
*P. falciparum* T2 mediated by anaemia	**−0.03**	**(−0.06**, **−0.01)**	−0.03	(−0.08, 0.01)	−0.02	(−0.05, 0.01)
*P. falciparum* T2 mediated by zinc	**0.04**	**(0.01, 0.07)**	**0.08**	**(0.01, 0.12)**	0.01	(−0.01, 0.03)

Boldface indicates statistical significance (*p* < 0.05). ^a^Model includes gender and baseline age. ^b^Data are presented as slope values for FM (95% CI) estimated across three data collection rounds growth curve models. ^c^Estimated indirect effects of *P. falciparum* on FM trajectory mediated by anaemia (α1^*^β1 in Figure 3) and changes in zinc concentrations (a2^*^b2).

**Figure 4:**
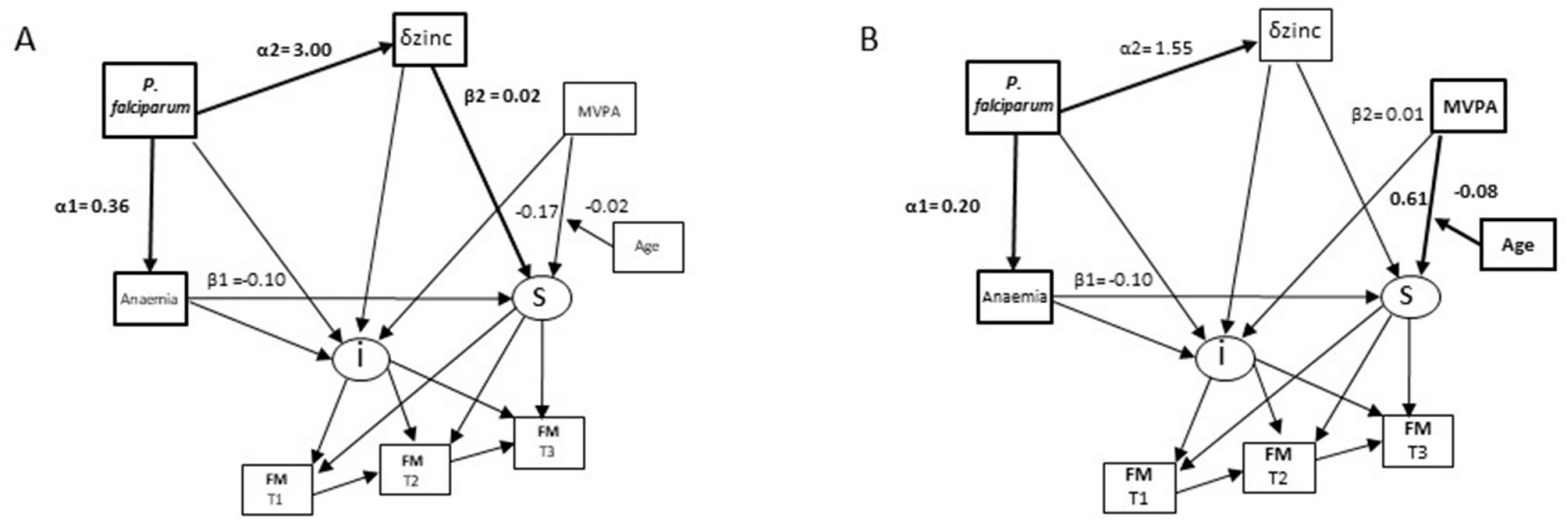
Differences in associations of micronutrient status, *P. falciparum* prevalence and child characteristics with changes in fat mass (FM) in Côte d‘Ivoire by sex. **(A)** boys. **(B)** girls.

Anaemia was not associated with the FM trajectory in the analysis stratified by sex. Changes in zinc concentrations were positively associated with the FM trajectory among boys (Table 2, Figure 4). *P. falciparum* infection at T2 had a strong positive association with zinc changes and so was indirectly associated with an increased FM trajectory (Table 2; α2^*^β2 in Figure 4). An interaction term between household food insecurity and household SES was again significantly associated with reductions in FM in the model for boys. Additionally, an interaction term between MVPA and age was associated with reduced FM among girls only suggesting that increased MVPA led to greater reductions in FM among older girls (Table 2; Figure 4).

### 3.4 Truncal fat free mass

More severe anaemia was again inversely associated with the trajectory of TrFFM overall and among girls similar to the pattern seen for FFM (Table 3). Vitamin A concentrations at T2 was again associated positively with the overall trajectory. *P. falciparum* infection at T2 again had an indirect inverse association with TrFFM trajectory overall and among girls, through its association with anaemia. *P. falciparum* infection again had an indirect inverse association with the TrFFM trajectory resulting from its inverse association with vitamin A concentrations. An interaction term between household food insecurity and household SES was again found to be significant only among boys indicating that the inverse association of food insecurity with TrFFM was reduced with greater household SES (Table 3).

**Table 3:** Associations of micronutrient status, household characteristics and *P. falciparum* prevalence with changes in truncal fat free mass (TrFFM) among 958 children in Côte d‘Ivoire overall and by sex.

	**Overall** ^**a**^		**Boys**		**Girls**	
	**TrFFM** ^b^	**95% CI**	**TrFFM**	**95% CI**	**TrFFM**	**95% CI**
**Change in TrFFM (T1–T3)** ^b^						
Anaemia T3	**−0.11**	**(−0.18**, **−0.05)**	−0.07	(−0.15, 0.02)	**−0.14**	**(−0.23**, **−0.05)**
Vitamin A concentrations T2	**0.01**	**(0.00, 0.02)**	0.01	(−0.01, 0.02)	0.01	(−0.02, 0.20)
Household food insecurity	0.21	(−0.25, 0.67)	−0.73	(−1.56, 0.12)	0.42	(−0.14, 1.04)
Household SES	0.16	(−0.30, 0.62)	**−1.03**	**(−1.99**, **−0.06)**	0.40	(−0.11, 0.93)
Food insecurity^*^SES	−0.04	(−0.20, 0.12)	**0.35**	**(0.02, 0.67)**	−0.11	(−0.29, 0.05)
***P. falciparum*** **effect on micronutrient mediators**						
**Anaemia T3**						
*P. falciparum* T2	**0.20**	**(0.06, 0.31)**	0.08	(−0.11, 0.28)	**0.26**	**(0.11, 0.42)**
**Vitamin A concentrations T2**						
*P. falciparum* T2	**−1.12**	**(−2.10**, **−0.16)**	−0.81	(−2.35, 0.73)	**−1.28**	**(−2.50**, **−0.05)**
**Indirect effects of** ***P. falciparum*** **on TrFFM**^c^						
Malaria mediated by anaemia	**−0.02**	**(−0.37**, **−0.01)**	−0.06	(−0.02, 0.08)	**−0.04**	**(−0.07**, **−0.01)**
Malaria mediated by vitamin A	**−0.01**	**(−0.05**, **−0.01)**	−0.01	(−0.03, 0.01)	0.01	(−0.03, 0.01)

Boldface indicates statistical significance (*p* < 0.05). ^a^Model includes gender and baseline age. ^b^Data are presented as slope values for TrFFM (95% CI). ^a^Trajectories estimated across three data collection rounds using latent growth curve models. ^c^Estimated indirect effects of *P. falciparum* on FFM trajectory mediated by anaemia and vitamin A.

### 3.5 Truncal fat mass

More severe anaemia was again found to be inversely associated with the TrFM trajectory in the overall model but now among boys in the models stratified by sex (Table 4). *P. falciparum* infection also had an indirect effect on this trajectory through its positive association with anaemia in the overall model. The indirect association with anaemia among boys or girls was not significant through this pathway. A small but positive association of the food insecurity—SES interaction term with TrFM was found only among boys. The interaction term between MVPA and age was also associated with TrFM among girls (Table 4).

**Table 4:** Associations of micronutrient status, child characterstics with changes in truncal fat mass/TrFM among 958 children in Côte d‘Ivoire^a^.

	**Overall** ^**a**^		**Boys**		**Girls**	
	**TrFM** ^b^	**95% CI**	**TrFM** ^b^	**95% CI**	**TrFM** ^b^	**95% CI**
**Change in TrFM (T1–T3)** ^b^						
Anaemia T2	**−0.07**	**(−0.11**, **−0.02)**	**−0.06**	**(−0.12**, **−0.01)**	−0.05	(−0.12, 0.14)
Zinc concentrations at T1	0.01	(−0.02, 0.02)	0.01	(−0.01, 0.02)	0.01	(−0.01, 0.02)
Changes in zinc concentrations T1–T3	0.01	(−0.01, 0.02)	0.01	(−0.01, 0.02)	0.01	(−0.02, 0.01)
Vitamin A concentrations T3	0.02	(0.01, 0.13)	0.01	(−0.01, 0.02)	0.01	(−0.03, 0.02)
Household food insecurity	−0.09	(−0.25, 0.67)	−0.50	(−1.03, 0.05)	0.07	(−0.40, 0.54)
Household SES	−0.06	(−0.30, 0.62)	**−0.64**	**(−1.25**, **−0.03)**	0.10	(−0.31, 0.52)
Food insecurity^*^SES	0.03	(−0.20, 0.12)	**0.22**	**(0.02, 0.43)**	−0.02	(−0.16, 0.12)
Age	**0.10**	**(0.06, 0.13)**	**0.05**	**(0.01, 0.10)**	**0.13**	**(0.09, 0.17)**
MVPA	0.08	(−0.16, 0.30)	−0.13	(−0.42, 0.16)	**0.35**	**(0.01, 0.70)**
Age^*^MVPA	−0.01	(−0.04, 0.019	0.02	(−0.02, 0.05)	**−0.04**	**(−0.08**, **−0.01)**
***P. falciparum*** **effect on micronutrient mediators**						
**Anaemia**						
Malaria T2	**0.28**	**(0.14, 4.36)**	**0.34**	**(0.12, 0.60)**	**0.21**	**(0.21, 0.41)**
**Change in zinc concentrations**						
Malaria T2	**1.20**	**(0.71, 3.16)**	**2.70**	**(0.55, 4.82)**	−0.33	(−2.24, 1.60)
**Indirect effects of** ***P. falciparum*** **on TrFM**^c^						
Malaria mediated by zinc changes anaemia	0.01	(−0.03, 0.01)	0.01	(−0.01, 0.02)	−0.01	(−0.02, 0.01)
Malaria mediated by anaemia	**−0.02**	**(−0.04**, **−0.01)**	−0.02	(−0.04, 0.01)	−0.01	(−0.03, 0.01)

Boldface indicates statistical significance (*p* < 0.05). ^a^Model includes gender and baseline age. ^b^Data are presented as slope values for TrFM (95% CI) estimated across three data collection rounds growth curve models. ^c^Estimated indirect effects of *P. falciparum* on TrFM trajectory mediated by anaemia and changes in zinc concentrations.

## 4 Discussion

This study has found that more severe anaemia was inversely associated with the trajectories of all four of the body composition outcomes while vitamin A concentrations and zinc concentrations changes were positively associated with these trajectories. *P. falciparum* infection plays a major role in determining these outcomes through its association with anaemia especially among girls. Additional differences were found between the sexes with food insecurity associated with reduced FFM among boys in lower SES households while increased MVPA led to greater reductions in FM among older girls. Reducing *P. falciparum* infection and anaemia, improving household food security and promotion of PA may improve child wellbeing and long-term health in Côte d'Ivoire through body composition improvements. School-based intervention trials assessing effects of PA promotion and nutrition supplementation could clarify these relationships and so inform policies and programs in Côte d'Ivoire concerned with reducing under nutrition and preventing overweight and obesity.

The association of anaemia with reduced FFM and TrFFM among school-aged children is a novel finding. Community based studies have found that iron deficiency are related to lower muscle mass among adults (13, 37). Iron deficiency has been shown to reduce myoblast proliferation, leading to muscle loss and atrophy (38). Reductions in FFM and TrFFM resulting from the effect of *P. falciparum* infection on anaemia is supported by reports of reductions in anaemia after implementation of malaria control mechanisms (21). The *P. falciparum*-anaemia association found only among girls contrasts with reports that males exhibit a higher malaria prevalence in endemic areas (39).

The increase in FFM associated with vitamin A concentrations could reflect the role of vitamin A and retinoic acid in promoting myogenesis. Animal models have reported that the administration of vitamin A administration to neonatal calves promoted myogenesis and postnatal muscle growth by promoting and increasing satellite cell density, accompanied with a shift to oxidative muscle fibers (40). Additionally, vitamin A deficiency is associated with decreased skeletal muscle performance although it is not clear if this may result from reduced myogeneseos (41). The association of *P. falciparum* infections with reduced vitamin A concentration, which leads to reduced FFM, may relate to findings that malaria infection is associated with reduced retinol concentrations even in asymptomatic infections (42). It is not clear if this relationship is causal since vitamin A can reduce severity of malaria (43).

The inverse association found between anaemia and FM measures may relate to studies reporting associations between obesity and anaemia (44). Increased body fat and visceral fat mass is positively associated with iron deficiency among preadolescents (45). Such low serum iron concentrations among obese subjects may result from low iron intake and low iron bioavailability (46). However, deficiency may also result from chronic inflammation caused by excessive adiposity rather than dietary factors (47). Iron deficiency anaemia may also increase obesity risk by decreasing thermogenic energy expenditure (48). It is not clear how association of *P. falciparum* infection with anaemia may contribute indirectly to reduced FM.

Increased zinc concentrations were positively associated with the overall FM trajectory and the trajectory among boys. Zinc supplementation of mice has been found to increase body fat percentage and visceral adipose tissue (49). However, zinc deficiency has been reported to be associated with increased fat accumulation and obesity among adults and children while zinc supplementation has been found to increase FFM (50). The positive effects of zinc supplementation on FFM is especially apparent among children with pre-existing growth failure (51). The positive association of *P. falciparum* infection with increased zinc concentrations may reflect zinc's role in *P. falciparum* growth cycle within erythrocyte while depletion inhibits growth (52). Few studies have examined the effect of malaria on FM through this pathway.

The positive association of vitamin A with changes in FM is not consistent with previous studies reporting that reduced serum vitamin A concentrations are associated with excess body adiposity while higher carotenoid concentrations are associated with lower adiposity (11, 53). The inverse association of anaemia found with vitamin A concentrations is supported by reports that vitamin A supplementation increases Hb levels and reduces the prevalence of anaemia (54). Anaemia may partly result from reduced iron mobilization among vitamin A deficiency individuals making it less available for erythropoiesis (55).

Household food insecurity was associated with reduced FFM, FM and TrFFM among boys from lower SES household. Lee reported that lower food security is linked to lower muscle mass among Korean men < 60 years of age (56). Severe food insecurity is also associated with muscle loss among older individuals in LMICs (57). It is not clear why this association was found only among boys.

Increased MVPA was associated with reductions in FM and TrFM only among older girls in our study. Previous studies have reported FM reductions among school-age children who were assigned to elevated PA group compared to a the control group participating in standard PA (58). Results from a cohort study reported that each minute/day of accelerometer-measured MVPA was associated with a 2.8 g decrease in children's fat mass (59). We reported previously that PA promotion among South African girls in the KaziAya project led to reduced FM and TrFM (60).

Our study is subject to a number of limitations. The loss of children to follow-up may have led to bias results due to differences between these children and children who remained. The smaller sample size is another limitation since it reduce statistical power. The study also could not address how the pandemic disrupted household food supply and reduced PA of housebound children. Additionally, the results are from children living in peri-urban settings and so have limited generalizability in rural settings or in wealthier South African student populations. Finally, the associations reported on in this paper may not necessarily be causal and so should be interpreted with some caution.

This study has several strengths, the FIML approach estimates a likelihood function for each individual based on the variables that are present producing unbiased parameter estimates and standard errors and so allows the use of the full sample in all analyses (61). Also, the LGCM framework requires smaller sample sizes when modeling complex representations compared to classical repeated measures analytic tools (62). It also provides flexibility in the analysis of the structural relationships between the independent variables and changes in body composition and so can model complex correlates of these changes.

## 5 Conclusions

This study has found that the high prevalence of *P. falciparum* in this population had important negative effects on muscle mass through its effects on anaemia among girls. Food insecurity was associated with reduced muscle mass in low SES households among boys while increased PA was associated with reduced fat mass among girls. Public health strategies to reduce the high prevalence of malaria in Côte d'Ivoire through the introduction of malaria vaccines and the implementation of policies that improve household food security could lead to relatively short term improvements in body composition and overall child health and wellbeing. The implementation of school-based programs promoting PA and improved nutrition would contribute both to these improvements while simultaneously contributing to longer-term health in adolescence and long-term risk of chronic metabolic diseases (63).

## Data Availability

The raw data supporting the conclusions of this article will be made available by the authors, without undue reservation.
