# Supplementary material for: Micronutrient status, food security, anaemia, Plasmodium infection, and physical activity as predictors of primary schoolchildren's body composition in Côte d'Ivoire
Source: Front Nutr. 2025 Jan 29;11:1524810. doi: 10.3389/fnut.2024.1524810 (PMC11816671; doi:10.3389/fnut.2024.1524810)
Supplement: Supplementary file 1 [file Table_1.docx]

**Supplementary Table 1.** Child characteristics at baseline in the Taabo, Côte d`Ivoire component of the

KaziAfya project

| Child characteristics | Overall | Boys | Girls |  |
| --- | --- | --- | --- | --- |
|  | Mean (SD) | Mean (SD) | Mean (SD) | *p*-value^a^ |
| Age (years) | 8.35 (1.75) | 8.37 (0.07) | 8.45 (0.07) | 0.98 |
| **Anthropometric measures** |  |  |  |  |
| Height (cm) | 123.65 (10.73) | 123.45 (0.46) | 123.80 (0.51) | 0.51 |
| Weight (kg) | 23.37 (5.46) | 23.16 (0.21) | 23.48 (0.27) | 0.25 |
| BMI (kg/m^2^) | 15.09 (1.45) | 15.06 (0.05) | 15.09 (0.07) | 0.57 |
| **Body composition** |  |  |  |  |
| FM (kg) | 4.56 (1.37) | **4.18 (0.04)** | **4.92 (0.07)** | **< 0.01** |
| FFM (kg) | 18.76 (4.25) | 18.98 (0.18) | 18.56 (0.21) | 0.13 |
| TrFM (kg) | 2.01 (0.61) | **1.92 (0.02)** | **2.10 (0.03)** | **< 0.01** |
| TrFFM (kg) | 11.95 (2.24) | **12.27 (0.08)** | **11.64 (0.11)** | **< 0.01** |
| **Categories of malnutrition** | *n (%)* | *n (%)* | *n (%)* |  |
| Stunted | 63 (12) | 33 (12.20) | 30 (11.76) | 0.87 |
| Wasted | 44 (9.42) | 19 (7.95) | 25 (10.96) | 0.26 |
| Overweight | 16 (3) | 7 (43.75) | 9 (56.25) | 0.54 |
