# Supplementary figures and images for: Micronutrient status, food security, anaemia, Plasmodium infection, and physical activity as predictors of primary schoolchildren's body composition in Côte d'Ivoire
Source: Front Nutr. 2025 Jan 29;11:1524810. doi: 10.3389/fnut.2024.1524810 (PMC11816671; doi:10.3389/fnut.2024.1524810)

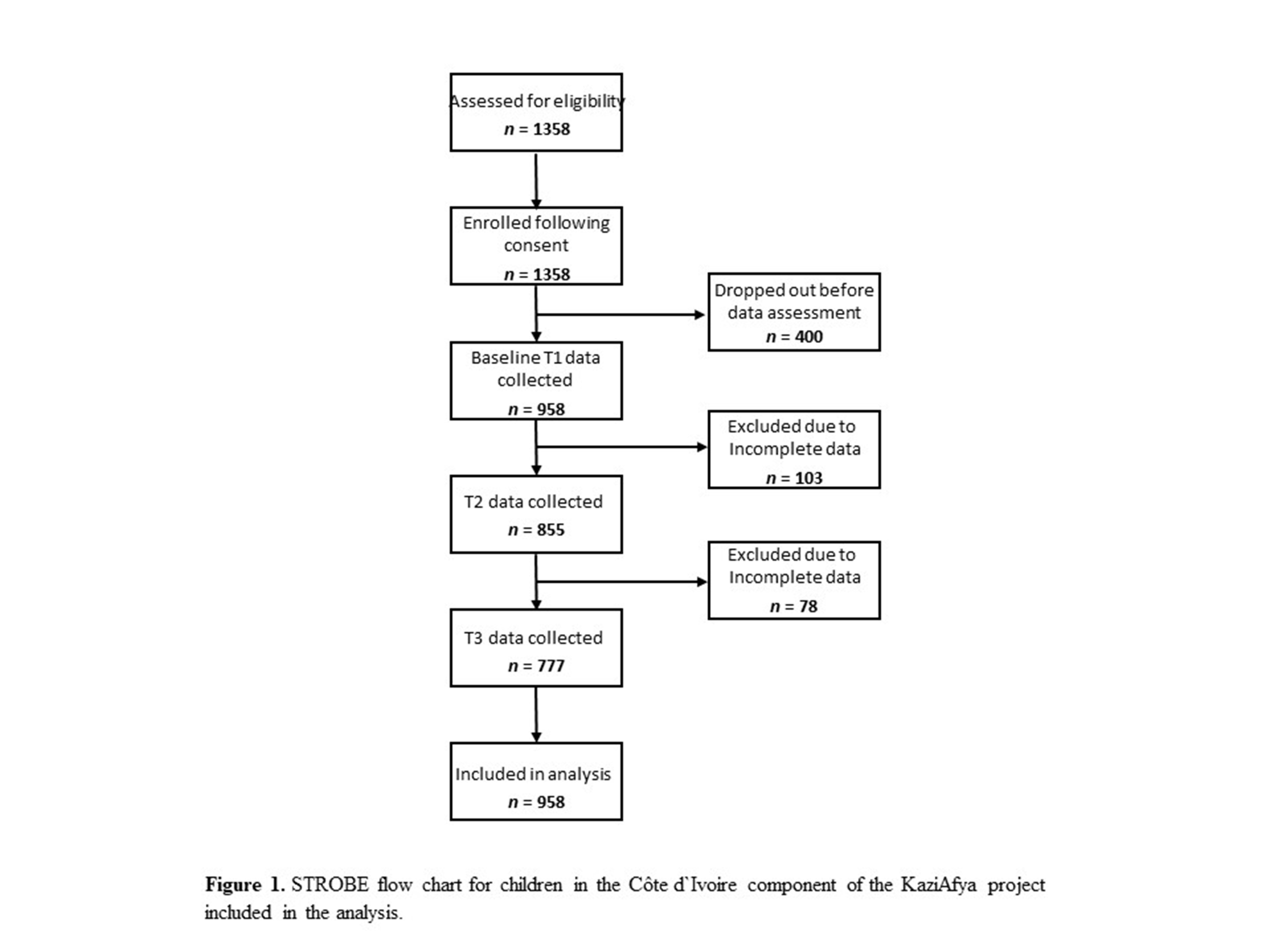

Supplement: Supplementary file 2 [file Image_1.jpeg]
